# Associations of PM_2.5_ and Black Carbon with Hospital Emergency Room Visits during Heavy Haze Events: A Case Study in Beijing, China

**DOI:** 10.3390/ijerph14070725

**Published:** 2017-07-05

**Authors:** Fengchao Liang, Lin Tian, Qun Guo, Dane Westerdahl, Yang Liu, Xiaobin Jin, Guoxing Li, Xiaochuan Pan

**Affiliations:** 1 Department of Occupational and Environmental Health, School of Public Health, Peking University, Beijing 100191, China; liangfengchao126@126.com (F.L.); tianlin510@foxmail.com (L.T.); guoqun1990123@163.com (Q.G.); kingxb@bjmu.edu.cn (X.J.); liguoxing@bjmu.edu.cn (G.L.); 2 School of Energy and Environment, City University of Hong Kong, Hong Kong, China; danewest03@gmail.com; 3 Department of Environmental Health, Rollins School of Public Health, Emory University, Atlanta, GA 30322, USA; yang.liu@emory.edu

**Keywords:** haze, PM_2.5_, BC, emergency room visits, population health

## Abstract

In January 2013, severe haze events over northeastern China sparked substantial health concerns. This study explores the associations of fine particulate matter less than 2.5 μm (PM_2.5_) and black carbon (BC) with hospital emergency room visits (ERVs) during a haze season in Beijing. During that period, daily counts of ERVs for respiratory, cardiovascular and ocular diseases were obtained from a Level-3A hospital in Beijing from 1 December 2012 to 28 February 2013, and associations of which with PM_2.5_ and BC were estimated by time-stratified case-crossover analysis in single- and two-pollutant models. We found a 27.5% (95% confidence interval (CI): 13.0, 43.9%) increase in respiratory ERV (lag02), a 19.4% (95% CI: 2.5, 39.0%) increase in cardiovascular ERV (lag0), and a 12.6% (95% CI: 0.0, 26.7%) increase in ocular ERV (lag0) along with an interquartile range (IQR) increase in the PM_2.5_. An IQR increase of BC was associated with 27.6% (95% CI: 9.6, 48.6%) (lag02), 18.8% (95% CI: 1.4, 39.2%) (lag0) and 11.8% (95% CI: −1.4, 26.8%) (lag0) increases for changes in these same health outcomes respectively. Estimated associations were consistent after adjusting SO_2_ or NO_2_ in two-pollutant models. This study provides evidence that improving air quality and reducing haze days would greatly benefit the population health.

## 1. Introduction

Beijing, the capital of China, experienced several severe haze episodes in January 2013 that focused attention on the issues of poor air quality. There has been a growing concern in this region that poor air quality is affecting both human health and the environment. Haze events mainly occur during winter months due to stable meteorological conditions and emissions from coal-fired power plants and residential heating [1,2]. Among the constituents of urban haze are aerosols. The fine particle fraction of these aerosols is known as PM_2.5_ (particles less than 2.5 µm in aerodynamic diameter), which is one of the main pollutants in haze [3]. The annual mean concentration of ambient PM_2.5_ in Beijing was 89.5 μg/m^3^ in 2013, which was much higher than both the annual level guided by the national air quality standard of China (National Ambient Air Quality Standard, NAAQS, http://kjs.mep.gov.cn/, 35 μg/m^3^) and the average level of whole mainland of China (72 μg/m^3^) according to the published statistics of Beijing Municipal Environmental Monitoring Center (http://www.bjepb.gov.cn/bjepb/323474/324034/324735/index.html). January 2013 was a month when significantly high periods of PM_2.5_ concentration occurred in Beijing, with a monthly mean concentration of 240 μg/m^3^ [4] and 77.3% days exceeding the serious level of the Air Quality Index [5]. 

Previous studies have reported associations between exposure to PM_2.5_ and adverse health effects worldwide, including increased cause-specific hospitalization and mortality [6,7] and reduced human lung [8] or immunologic function [9]. There are also studies reporting the impact of PM_2.5_ on endocrine dysfunction [10] and adverse pregnancy outcomes [11]. Saxena et al. [12] reported that persons travelling in highly polluted areas have higher levels of sub-clinical ocular surface changes in Delhi. Associations between PM_2.5_ exposure and conjunctivitis morbidity were reported in Canada [13] and Japan [14]; Torricelli’s study [15] in Brazil found that increased PM_2.5_ was associated with the decrease in tear osmolality, which indicated that exposure to PM_2.5_ may also affect human ocular adnexal (e.g., lacrimal apparatus). Currently, studies on the ocular hazardous effects of PM_2.5_ are still very limited, especially in China. On the other hand, premature death counts and the economic cost of death due to PM_2.5_ exposure attributed to haze pollution events have been reported [16,17]; however, most of these use the exposure-response associations observed on non-haze days. Quantitative links between PM_2.5_ exposure and its related health effect at high concentrations (e.g., haze days) have not been well-characterized because pollutant and health data from places with such conditions as China are seldom available. Thus, while considerable evidence links PM_2.5_ to the occurrence and development of various diseases, there still remain challenges and opportunities for studies of PM_2.5_ associated health effects in China.

One important aerosol component of haze is combustion-derived black carbon (BC). As a component of PM_2.5_ [18], it has been associated with increased inflammatory/endothelial response [19], elevated plasma total homocysteine [20], decreased heart rate variability and heart rate deceleration capacity [21]. Previous studies reported positive associations between BC exposure and human respiratory and cardiovascular mortality, cardiovascular hospitalization [22] and blood pressure in healthy adults [23]. Gold et al. [24] found that elevated BC also predicted increased risk of ST-segment (a phase of the cardiac action potential) depression in elder subjects. Wang et al. [25] reported that both PM_2.5_ and BC were associated with total emergency-room visits in Shanghai. Janssen et al. [26] performed a meta-analysis to evaluate BC as an additional indicator in air particulate matter associated health risks assessment. They found the extension of life expectancy with the reduction of BC was four to nine times higher than the benefit of an equivalent change in PM_2.5_ mass, but their measured effects on all-cause and cardiovascular mortality of an interquartile range (IQR) increase were similar. Thus, BC may play an important role in PM_2.5_ related health risks, which need more epidemiological evidences. However, BC data is not widely available since it is not included in routine air monitoring programs.

This study was conducted to explore the links of PM_2.5_ and BC with cause-specific hospital emergency room visits (ERVs) during extreme haze events in Beijing using a time-stratified case-crossover strategy. Different lag periods were included to detect the potential delayed or cumulative effects of exposure. Data from other available gaseous pollutants were also included. Stratification analyses for age and gender were performed to determine whether these subgroups demonstrated differing responses.

## 2. Materials and Methods

### 2.1. Health Data

Daily hospital emergency room visits from 1 December 2012 to 28 February 2013 were collected from the Peking University Third Hospital, the emergency center that serves unscheduled cases living around it and other areas in Beijing. As one of the top-level hospitals, over 10 thousand emergency visits were recorded every month. During the clinical process, the information of each visit was recorded on a standardized form, including an identification number, gender, age and primary diagnoses. Then a database was built based on these cases. The primary diagnosis were coded by medically trained researchers according to the International Classification of Diseases, 10th Revision (ICD-10) [27]. Respiratory diseases (Res., ICD-10: J00-J99), cardiovascular diseases (Car., ICD-10: I00-I99), and ocular diseases (Ocu., ICD-10: H00-H59, including diseases of eye and associated adnexa) were selected for analysis; emergency admissions for trauma were excluded. 

### 2.2. Exposure Data

PM_2.5_, sulfur dioxide (SO_2_), and nitrogen dioxide (NO_2_) data from 12 ambient air quality monitoring sites were obtained from China Municipal Environmental Monitoring Center and processed to produce 24 h daily records from 27 November 2012 to 28 February 2013. Daily mean temperature (Tem.) and relative humidity (RH) from one station (located at N39°48′, E116°28′) during the study period were obtained from the China Meteorological Data Sharing Service System. 

Black Carbon (BC) monitoring was conducted using an Aethalometer (Model AE 21, Magee Scientific Company, Berkeley, CA, USA) at one site only located on the campus of Health Science Center of Peking University, which is about 450 m away from Peking University Third Hospital. This monitor was the only source of BC data available for this study. The machine uses a continuous filtration and optical measurement method to give real-time data of BC. In this study, we used 5-min average values and computed the daily average concentration for data analysis. There were 11.7% daily BC values missing during this period. As daily average PM_2.5_ and BC was highly correlated (correlation coefficient = 0.92) in our study period, we built a simple regression model to fill in missing data for BC. The regression and fitted parameters were provided as follows:

$$\hat{C_{BC}}=0.044\times C_{PM2.5}-0.046$$

where, $\hat{C_{BC}}$ is the predicted daily concentration of BC and $C_{PM2.5}$ is the observed PM_2.5_ values of the corresponding day that BC is missing.

**Figure 1:**
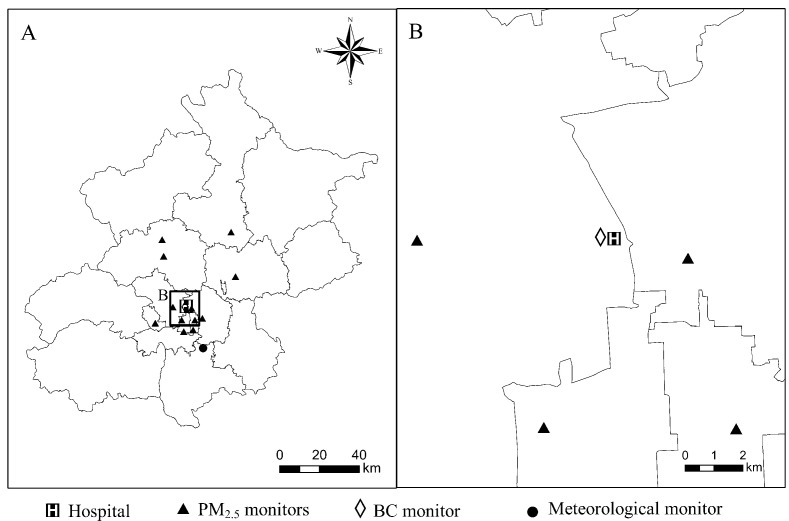
Spatial locations of target hospital and ground PM_2.5_, BC (black carbon), meteorological monitors in this study. The entire region of study is shown in panel **A** and the area nearest the hospital is shown in panel **B**.

Locations of Peking University Third Hospital, PM_2.5_, BC and meteorological monitors are shown in Figure 1.

### 2.3. Statistical Analysis

The case-crossover design with a time-stratified approach was used to estimate the impacts of short exposures on the acute health outcomes. Each subject served as their own control. Exposure levels at a time period just before hospital emergency visits were compared with those at other times when he or she was not hospitalized, thus time trend and unvarying factors such as age, sex, smoking, nutrition status, and socio-economic conditions were controlled by this design [28,29]. In this study, the associations between PM_2.5_ as well as BC and hospital emergency admissions during a haze season were estimated using conditional logistic regression model in SAS (version 9.3, SAS institute Inc., Cary, NC, USA). The case period was from 1 December 2012 to 28 February 2013 (i.e., a three month period) and the days with the same day-of-week in the same calendar month as the case days were chosen as the control. In single-pollutant models, public holiday, temperature, and relative humidity were adjusted. In the case of temperature, previous research has shown that the 15-day moving average (lag0–14) temperature had larger effects on human health [30,31]. Thus, we used temperature and relative humidity at lag0–14 in the model simultaneously. Then, daily (24 h) concentration of SO_2_ or NO_2_ were added in two-pollutant models, respectively, to avoid the multicollinearity between them. In these models, the percentage increases in cause-specific ERVs for an interquartile range (IQR) increase in PM_2.5_ and BC in the single lag models were estimated. Lag days ranging from 0 to 4 and moving average from 2 days (01) up to 5 days (04) were evaluated.

The hospital serves the entire region, so in this study we used the averaged concentration levels of PM_2.5_, SO_2_, and NO_2_ from all 12 monitors in the Beijing region shown instead of just the monitors near the hospital. Analyses were performed to find the lag period with the largest associations between SO_2_ and NO_2_ and ERVs to minimize the bias introduced by them. Both SO_2_ and NO_2_ showed a largest association with ERVs at lag2 in single-pollutant models. Our study was conducted during the winter season in Beijing, thus pollen was not likely to be an important factor that would bias the associations between PM_2.5_ and BC and ERVs, especially for ocular diseases, so pollen levels were not considered in the models.

We also did stratification analysis of age, gender and PM_2.5_ levels in single-pollutant models. The lag period at which the associations between PM_2.5_ and BC and cause-specific ERVs were largest and also significant in the unstratified models were chosen as the lag day of pollutants in this analysis. If no significant associations were found, the largest ones were chosen. 

To evaluate the sensitivity of imputing missing BC data, we compared the results obtained using the original dataset with missing values to those from the imputed dataset. Based on Winquist’s [32] study, we also performed a sensitivity analysis by adding quadratic and cubic terms of PM_2.5_ or BC to evaluate the adequacy of using linear pollutant terms in the single-pollutant models. Combined effects of linear, quadratic and cubic terms for an IQR increase in PM_2.5_ or BC were calculated and compared with the associations estimated with only linear terms in the models.

## 3. Results

**Figure 2:**
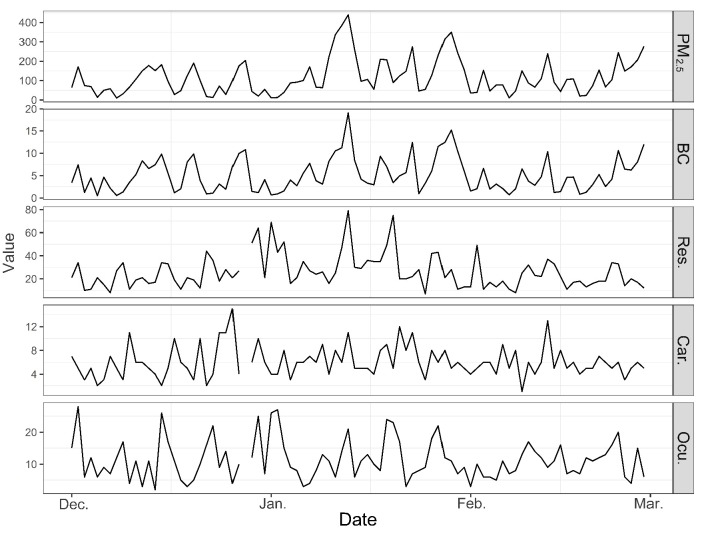
Time-series plot of daily PM_2.5_ and BC concentrations and cause-specific hospital emergency room visits (ERVs) during the study period.

There were 2323, 541, and 1007 respiratory, cardiovascular, and ocular disease case admissions collected and analyzed the during study period, respectively. The male proportion for respiratory, cardiovascular and ocular ERV cases were 39.3%, 48.3% and 44.4% respectively; 91.4%, 56.0% and 91.9% of the cases were younger than 65 years old, respectively. Table 1 shows the summary statistics for daily ERV counts of respiratory, cardiovascular and ocular diseases, air pollutants and meteorological variables during the study period. The average daily level of PM_2.5_ and BC were 119.8 and 5.2 μg/m^3^ respectively. Time-series plots for PM_2.5_, BC and ERV counts during the study period are shown in Figure 2. BC concentrations are in good temporal agreement with PM_2.5_. High daily concentrations of PM_2.5_ of 53 days exceed secondary daily standard of NAAQS (75 μg/m^3^).

Spearman correlation coefficients (r) between air pollutants, meteorological variables and daily respiratory, cardiovascular and ocular diseases ERV counts are shown in Table 2. Positive and strong correlations were observed between PM_2.5_ and BC (r = 0.920, *p* < 0.01), SO_2_ (lag2) and NO_2_ (lag2) (r = 0.914, *p* < 0.01). Both PM_2.5_ and BC were positively correlated with daily respiratory and cardiovascular ERVs counts and weakly negatively correlated with ocular ERVs counts (r were −0.099 and −0.123, respectively) with no statistical significance found (*p* > 0.05).

**Figure 3:**
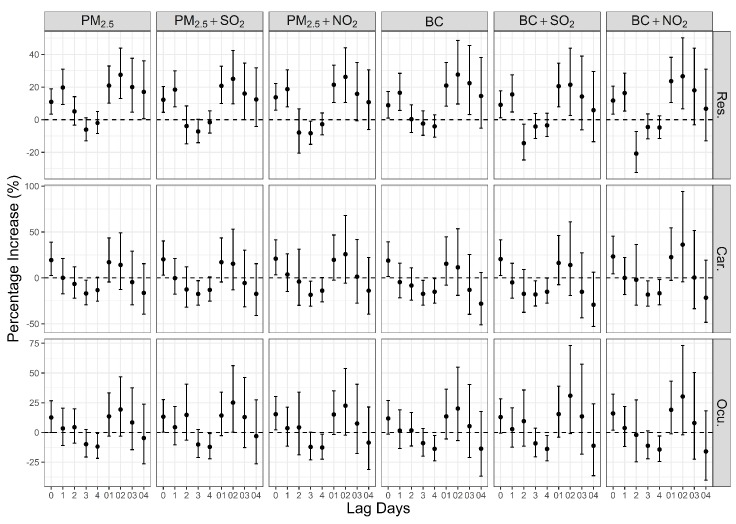
Estimated percentage increase in hospital ERVs (95% CI) for an interquartile range (IQR) increase in PM_2.5_ and BC using the single- and two-pollutant models.

Figure 3 shows the lag structures of associations of an IQR increase in PM_2.5_ (121.6 μg/m^3^) and BC (5.4 μg/m^3^) from lag0 to lag4 and moving average from lag01 to lag04 with respiratory, cardiovascular, and ocular ERVs. Significant associations were found between both PM_2.5_ and BC and respiratory ERVs on lag0, lag1, lag01–02, with a largest one at lag02, even after adjusting for SO_2_ and NO_2_. For an IQR increase in PM_2.5_, the largest estimated percent increases in respiratory ERVs were observed at lag02 with a value of 27.5% (95% confidence interval (CI): 13.0, 43.9%) in the single-pollutant model and values of 25.0% (95% CI: 9.7, 42.4%) and 26.2% (95% CI: 10.6, 44.1%), respectively, after adjusting SO_2_ and NO_2_; for an IQR increase in BC, the largest estimated percent increases in respiratory ERVs also appeared at lag02, which were 27.6% (95% CI: 9.6, 48.6%), 21.4% (95% CI: 2.6, 43.8%), and 26.6% (95% CI: 6.7, 50.2%), respectively in the single- and two-pollutant models. Both PM_2.5_ and BC were significantly and positively associated with cardiovascular ERVs occurred at lag0, which were 19.4% (95% CI: 2.5, 39.0%), 20.2% (95% CI: 3.0, 40.2%) and 20.9% (95% CI: 3.4, 41.4%), respectively, for PM_2.5_ before and after adjusting SO_2_ and NO_2_, and 18.8% (95% CI: 1.4, 39.2%), 20.5% (95% CI: 2.6, 41.5%) and 23.3% (95% CI: 4.5, 45.5%), respectively, for BC. The associations between PM_2.5_ and ocular ERVs were positive and significant at lag0 in both single- and two-pollutant models, which were 12.6 (95% CI: 0.0, 26.7%), 13.2 (95% CI: 0.3, 27.6%) and 15.3 (95% CI: 2.1, 30.2%), respectively. A significant association was found between BC and ocular ERVs at lag0 after adjusting NO_2_ (16.0% (95% CI: 1.8, 32.2%)) (*p* < 0.05).

**Figure 4:**
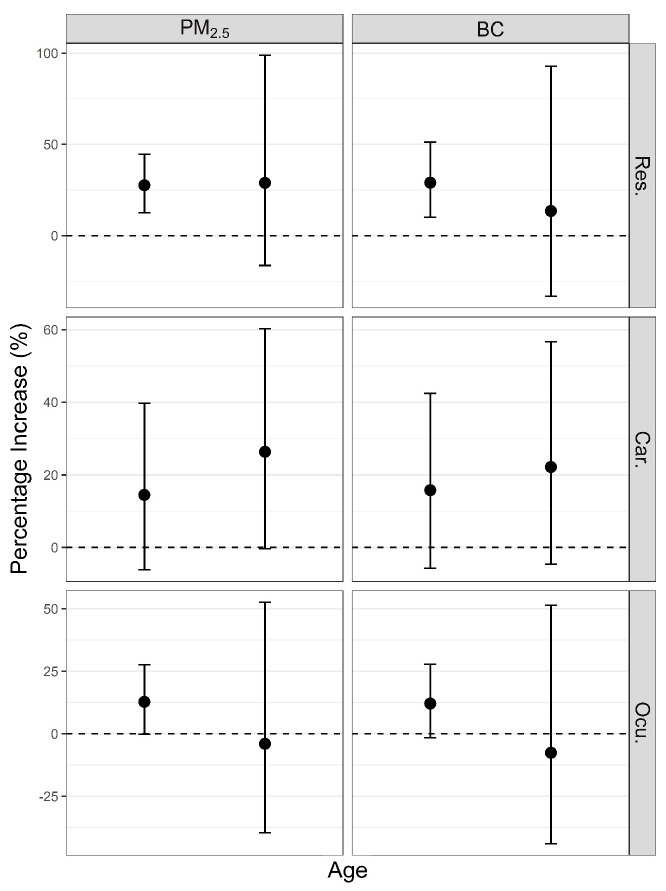
Estimated percentage increase in hospital ERVs (95% CI) for an IQR increase in PM_2.5_ and BC in different age groups.

Associations between PM_2.5_ and BC and respiratory ERVs at lag02, cardiovascular and ocular ERVs at lag0 were selected in the modified effects analysis. No significant differences were found between age groups for the associations in the three cause-specific ERVs, and larger 95% CIs were observed in elders (older than 65) than that in younger subjects (younger than 65) (Figure 4). The estimated associations between PM_2.5_ and respiratory ERVs were similar in youngers individuals and elders, which were 27.6% (95% CI: 12.5, 44.7%) and 28.9% (95% CI: −16.4, 98.8%), respectively; a smaller association between BC and respiratory ERVs was seen in elders, which was 13.5% (95% CI: −33.2, 92.9%) compared to 29.0% (95% CI: 10.0, 51.3%) in youngers individuals. Both PM_2.5_ and BC were associated with larger increments in cardiovascular ERVs in elders (26.4% (95% CI: −0.4, 60.3%) and 22.2% (95% CI: −4.6, 56.7%), respectively) than that in youngers individuals (14.5% (95% CI: −6.2, 39.7%) and 15.8% (95% CI: −5.8, 42.4%), respectively); however, they were negatively associated with ocular ERVs in elders (−4.0% (95% CI: −39.6, 52.6%) and −7.6% (95% CI: −43.7, 51.5%), respectively) compared to positive associations of 12.8% (95% CI: −0.2, 27.6%) and 12.1% (95% CI: −1.6, 27.8%), respectively, in younger subjects. 

**Figure 5:**
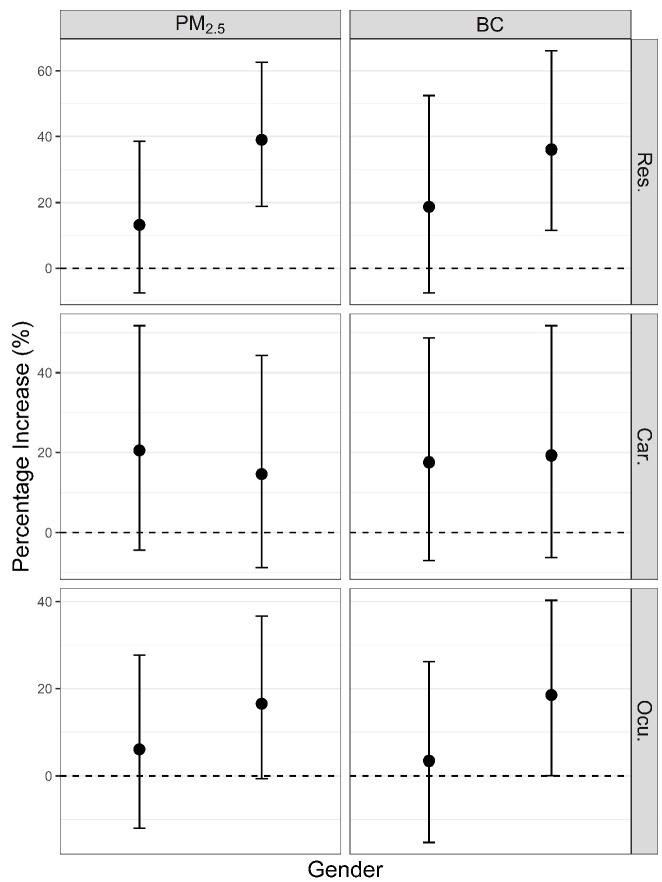
Estimated percentage increase in hospital ERVs (95% CI) for an IQR increase in PM_2.5_ and BC in males and females.

When examining effect modification by gender (Figure 5), we found that associations between elevated PM_2.5_ and BC concentrations and increments of respiratory and ocular ERVs were larger in females, whereas that was larger in males between PM_2.5_ and cardiovascular ERVs, though all these differences are not statistically significant. An IQR increase in PM_2.5_ was associated with 13.2% (95% CI: −7.5, 38.6%) and 39.0% (95% CI: 18.8, 62.6%) increases among males and females in respiratory ERVs, respectively, 20.5% (95% CI: −4.4, 51.8%) and 14.7% (95% CI: −8.8, 44.3%) increases in cardiovascular diseases, respectively, and 6.0% (95% CI: −12.0, 27.7%) and 16.5% (95% CI: −0.6, 36.7%) increases in ocular ERVs, respectively. Similar results were seen for associations between BC and ERVs, except that the estimated percentage increase in male ERVs of cardiovascular disease was slightly lower than that in female ERVs with an IQR increase in BC, which was 17.6% (95% CI: −0.6, 36.7%) compared to 19.3% (95% CI: −6.2, 51.8%).

Sensitivity analysis on associations between ERVs and BC before and after daily missing data imputed were shown in Table S1. No statistically significant differences were observed when using the original dataset with missing BC values and those from the imputed dataset. Table S2 (see Supplementary Materials) shows sensitivity analysis on the adequacy of linear terms of PM_2.5_ or BC that no significant differences were observed for the estimated associations without and with quadratic and cubic terms of PM_2.5_ or BC fitted in the single-pollutant models.

## 4. Discussion

In this study, we observed significant associations between PM_2.5_ and BC and hospital emergency visits for three specific health outcomes, including respiratory, cardiovascular and ocular diseases during a winter haze season in Beijing, China. To our knowledge, this is one of a few studies to investigate associations between PM_2.5_ and BC and hospital ERVs during the periods with high ambient pollutant levels, and the first study to explore particulate air pollutant links to ERVs related to ocular diseases in China. By using a time-stratified case-crossover method, potential confounders, such as age, sex, microenvironment, socioeconomic status, nutritional status, and personal habit were controlled. As China’s emergency departments in hospitals give timely service to patients with sudden and life-threatening diseases or excruciating symptoms, the observed occurrences of ERVs associated with PM_2.5_ or BC exposure were equivalent to the excess morbidity seen in the corresponding diseases. 

The 24 h average PM_2.5_ concentration during the study period was 119.8 μg/m^3^, which was much higher than the national secondary ambient air quality standard in China (daily 75 μg/m^3^). However, for BC ambient pollution, no standard levels have been set either in China or elsewhere in the world. Compared with other studies conducted to estimate the associations of PM_2.5_ or BC with hospital emergency visits outside of China [33], PM_2.5_ and BC levels were fairly high during the study period, with IQRs of 121.6 μg/m^3^ and 5.5 μg/m^3^, respectively. In the St. Louis metropolitan area of the U.S., an IQR increase in PM_2.5_ (11.1 μg/m^3^) and elemental carbon (0.42 μg/m^3^) were associated −0.1% (95% CI: −1.9, 1.6%) and 1.6% (95% CI: 0.2, 3.0%) increases of cardiovascular ERVs, respectively [33], whereas the increments in our study were 19.4% (95% CI: 2.5, 39.0%) and 18.8% (95% CI: 1.4, 39.2%) for an IQR increase in PM_2.5_ and BC. Baseline air pollution levels (i.e., IQRs) as well as factors such as ambient pollutant levels and population sensitivity and chemical composition of particulate matter may contribute to the large differences between these two studies. Smaller associations of PM_2.5_ were also reported in Beijing with ERVs in the same hospital as our study during 2007 to 2008, an IQR increase in PM_2.5_ (68 μg/m^3^) was associated with 3.1% (95% CI: 0.2, 6.0%) increment of cardiovascular ERVs in winter [6]. Thus, high levels of PM_2.5_ may lead to the increases of hospital ERVs in Beijing.

BC was positively associated with respiratory, cardiovascular and ocular ERVs in our study (Figure 3). The limited scientific evidence currently available showed that the health effects associated with an IQR increase of BC are similar but not exactly same with PM_2.5_, especially for cardiovascular effects. Zanobetti’s study [22] in Boston found that an increase in the difference between 90th and 10th percentile in PM_2.5_ (16.3 μg/m^3^) and BC (1.7 μg/m^3^) were associated with an 8.6% (95% CI: 1.2, 15.4%) increase and an 8.3% (95% CI: 0.2, 15.8%) increase in emergency myocardial infarction hospitalization, respectively. Wang et al. [25] reported that an IQR increase in PM_2.5_ and BC (42.2 μg/m^3^ and 2.7 μg/m^3^, respectively) were associated with a 1.88% (95% CI: 0.69, 3.06%) and a 1.33% (95% CI: 0.34, 2.32%) increase in emergency room visits, respectively in Shanghai. In our study, estimated ERVs associations with an IQR increase in exposure were similar for PM_2.5_ and BC (Figure 3). This finding is in agreement with similar studies in Shanghai and Boston. Although PM_2.5_ and BC were highly correlated and their associations with ERVs were similar, these findings call additional attention to BC monitoring and related health effects. Our study and that of Janssen’s [26] suggest that on a per μg basis, BC may be more toxic than generic PM_2.5_. Further studies using more robust data are needed to refine this point. Further, associations of PM_2.5_ and BC with cause-specific ERVs did not completely overlap in our study; PM_2.5_ associated with ERVs due to cardiovascular causes more closely in males, whereas a stronger association was seen between BC and cardiovascular ERVs in females, which suggests their effects on health may not be exactly the same. In addition, given the evidence that combustion-related components of particulate matter are more harmful than the non-combustion fractions [34], as a component of both fine and coarse particulate matter [18], BC may underlie some of the health impact of PM_2.5_. Further study is needed to clarify this. 

Currently, limited information is available on the associations of PM_2.5_ or BC with ocular disorders, especially in China. Previous studies indicated that PM_2.5_ may lead to the occurrence of ocular disorders by affecting human tear film stability and tarsal goblet cells density [15,35]. In our study, PM_2.5_ was significantly associated with ocular disorders on the same day with the clinical diagnosis (percentage increase: 12.6%; 95% CI: 0.0, 26.7%), which was consistent with the studies that PM_2.5_ significantly associated with outpatient attendance for allergic conjunctivitis (odds ratio = 9.05, *p* = 0.0463) in non-pollen season in Tokyo [14] and conjunctivitis ERVs (OR = 1.017, 95% CI: 1.003, 1.031) in nine cities in Ontario, Canada [36]. Though no evidence was found for BC and the morbidity of ocular diseases previously, our study indicated that BC also had a significant association with ocular disorders ERVs during highly polluted days (percentage increase: 16.0%; 95% CI: 1.8, 32.2%), after adjusting NO_2_ concentrations. 

In our study, associations of PM_2.5_ and BC with hospital ERVs appeared not to be confounded by NO_2_ or SO_2_; they varied little after adjusting 2-day lag concentration of SO_2_ or NO_2_. Compared to non-haze days, the increase of particulate matter was much more significant than that of SO_2_ and NO_2_ during haze days [37], which suggests independent associations of PM_2.5_ and BC with cause-specific ERVs in the highly polluted period of the study. We found the estimated associations between PM_2.5_ and BC and cardiovascular ERVs were larger in subjects older than 65 years. Similar results were seen where both PM_2.5_ mass and its chemical constituents are associated with ERVs for ischemic stroke among patients older than 65 years in Taiwan [38]. This group may be viewed as a special group with poor immune function and potential underlying cardiovascular diseases. However, we also found that positive associations between PM_2.5_ and BC and ocular ERVs were only found in younger subjects. The toxic actions of air pollutants on different parts of human body system and different age groups may differ. This may lead to the differences in response to PM_2.5_ or BC among age groups. Besides, the small number of elder subjects contribute to larger 95% CIs of associations. Further studies with more cases may help to detect clearer differences between age groups. The observations that females had stronger associations of PM_2.5_ or BC exposure with ERVs than males in our study with the exception of PM_2.5_ and cardiovascular ERVs suggests that factors such as hormonal levels, household duties and time spent in outdoor air may contribute to observed differences in response.

Some limitations of this study should be noted. Firstly, we only collected ERVs records from one hospital, which may introduce bias of detecting an association. Secondly, the pollutant exposures for these subjects were estimated from PM_2.5_ data representing a wide range of monitoring sites and BC data representing only one site. This may contribute to unknown degrees of exposure misclassification. Thirdly, a small portion of BC data was missing and an imputing method was used. Although results of sensitivity analysis suggest the estimation of the associations was not significantly affected by the imputation method (Table S1, see Supplementary Materials), this approach may introduce some uncertainty in the estimation of associations between BC and ERVs. This study was performed during the winter in Beijing, a season when people spend more time indoors than outdoors [39]; thus PM_2.5_ and BC exposure were estimated without accounting for the seasonal differences and differences in activity patterns. In addition, since there is a high correlation between PM_2.5_ and BC, it is difficult to separate independent effects for these individual pollutants. Finally, we considered only two facets of particulate air pollution. Other components of particulate matter may also be important. 

Our future work will focus on comparing the associations between ERVs and exposure of air pollutants by using varied methodologies (e.g., introducing techniques such as imputation modeling to estimate PM_2.5_ or BC exposure more accurately). This work will also attempt to locate and include data collected by others for BC in Beijing. With the continued improvement in the health data collection system and the expanding availability of air pollutants data, this study can be extended both temporally and spatially and the additional health effects due to severe haze exposure may be calculated. Further studies on non-linear associations of air pollution with ERVs and distinguishing PM_2.5_ and BC health effects are also proposed.

## 5. Conclusions

In conclusion, our study found that elevated levels of ambient PM_2.5_ as well as BC are associated with increases in hospital ERVs for respiratory, cardiovascular, and ocular diseases during severe haze in Beijing. It is possible that the findings of this study may be viewed as suggesting that BC contributes to the associations of PM_2.5_ with related health outcomes. To the extent that this is true, it may be health protective to establish regulations for BC. Our findings suggested that improving air quality and reducing haze days would benefit the population health. 

## Figures and Tables

**Table 1:** Descriptive statistics of daily environmental variables and hospital emergency admissions over a three month period. RH: relative humidity.

Variable	Mean	SD	Min.	Percentile			Max.
				25%	50%	75%	
Tem. (°C)	−3.5	3.0	−9.7	−5.3	−3.5	−1.6	5.0
RH (%)	53.7	19.4	19.0	38.0	52.5	69.5	91.0
PM_2.5_ (μg/m^3^)	119.8	96.3	10.2	49.3	96.3	170.9	439.3
BC (μg/m^3^)	5.2	3.8	0.5	2.1	4.2	7.5	19.1
SO_2_ (μg/m^3^)	56.8	28.6	10.1	37.6	51.5	76.8	131.0
NO_2_ (μg/m^3^)	68.5	30.6	12.6	44.9	69.8	89.3	144.1
Res. (counts)	26.1	14.8	7	16	21	34	79
Car. (counts)	6.1	2.6	1	5	6	8	15
Ocu. (counts)	11.3	6.2	2	7	11	15	28

BC: black carbon; Res.: respiratory diseases; Car.: cardiovascular diseases; Ocu.: ocular diseases.

**Table 2:** Coefficient for the relationship between daily environmental variables and hospital emergency admissions.

Variable	Tem. (lag0–14)	RH (lag0–14)	PM_2.5_ (lag0)	BC (lag0)	SO_2_ (lag2)	NO_2_ (lag2)	Res.	Car.	Ocu.
Tem. (lag0–14)	1.000								
RH (lag0–14)	−0.046	1.000							
PM_2.5_ (lag0)	−0.119	0.137	1.000						
BC (lag0)	−0.118	0.110	0.920 **	1.000					
SO_2_ (lag2)	−0.145	0.155	0.186	0.179	1.000				
NO_2_ (lag2)	−0.159	0.291 **	0.204	0.193	0.914 **	1.000			
Res.	−0.442 **	−0.019	0.033	0.032	0.183	0.164	1.000		
Car.	−0.220 *	0.090	0.171	0.169	−0.095	−0.029	0.080	1.000	
Ocu.	−0.078	−0.017	−0.099	−0.123	0.007	0.001	0.576 **	0.025	1.000

Note: Spearman rank correlation analysis, * *p* < 0.05, ** *p* < 0.01.

## Supplementary Materials

The following are available online at www.mdpi.com/1660-4601/14/7/725/s1, Table S1: Sensitivity analysis on the imputation for missing data of BC in the single-pollutant models. Table S2: Sensitivity analysis on the adequacy of linear terms of PM_2.5_ or BC in the single-pollutant models.

## References

[1] Levy Zamora M.E. Wintertime haze formation in Beijing. Proceedings of the AGU Fall Meeting Abstracts, AGU Fall Meeting.

[2] Zhang X.Y., Wang J.Z., Wang Y.Q., Liu H.L., Sun J.Y., Zhang Y.M. (2015). Changes in chemical components of aerosol particles in different haze regions in China from 2006 to 2013 and contribution of meteorological factors. Atmos. Chem. Phys..

[3] Tan J., Duan J., He K., Ma Y., Duan F., Chen Y., Fu J. (2009). Chemical characteristics of PM_2.5_ during a typical haze episode in Guangzhou. J. Environ. Sci..

[4] Li T.T., Du Y.J., Mo Y., Xue W.B., Xu D.Q., Wang J.N. (2013). Assessment of haze-related human health risks for four Chinese cities during extreme haze in January 2013. Zhonghua Yi Xue Za Zhi.

[5] Xie Y.B., Chen J., Li W. (2014). An assessment of PM_2.5_ related health risks and impaired values of Beijing residents in a consecutive high-level exposure during heavy haze days. Huan Jing Ke Xue.

[6] Su C., Breitner S., Schneider A., Liu L.Q., Franck U., Peters A., Pan X.C. (2016). Short-term effects of fine particulate air pollution on cardiovascular hospital emergency room visits: A time-series study in Beijing, China. Int. Arch. Occup. Environ. Health.

[7] Li M.H., Fan L.C., Mao B., Yang J.W., Choi A.M.K., Cao W.J., Xu J.F. (2016). Short-term exposure to ambient fine particulate matter increases hospitalizations and mortality in COPD: A systematic review and meta-analysis. Chest.

[8] Kannell W.B. (2004). The effect of air pollution on lung development from 10 to 18 years of age—NEJM. N. Engl. J. Med..

[9] Zhao J., Gao Z., Tian Z., Xie Y., Xin F., Jiang R., Kan H., Song W. (2013). The biological effects of individual-level PM(2.5) exposure on systemic immunity and inflammatory response in traffic policemen. Occup. Environm. Med..

[10] Brook R.D., Cakmak S., Turner M.C., Brook J.R., Crouse D.L., Peters P.A., Van D.A., Villeneuve P.J., Brion O., Jerrett M. (2013). Long-term fine particulate matter exposure and mortality from diabetes in Canada. Diabetes Care.

[11] Wilhelm M., Ritz B. (2005). Local variations in co and particulate air pollution and adverse birth outcomes in Los Angeles County, California, USA. Environ. Health Perspect..

[12] Saxena R., Srivastava S., Trivedi D., Anand E., Joshi S., Gupta S.K. (2003). Impact of environmental pollution on the eye. Acta Ophthalmol. Scan..

[13] Szyszkowicz M., Shutt R., Kousha T., Rowe B.H. (2014). Air pollution and emergency department visits for epistaxis. Clin. Otolaryngol..

[14] Mimura T., Ichinose T., Yamagami S., Fujishima H., Kamei Y., Goto M., Takada S., Matsubara M. (2014). Airborne particulate matter (PM_2.5_) and the prevalence of allergic conjunctivitis in Japan. Sci. Total Environ..

[15] Torricelli A.A., Novaes P., Matsuda M., Braga A., Saldiva P.H., Alves M.R., Monteiro M.L. (2013). Correlation between signs and symptoms of ocular surface dysfunction and tear osmolarity with ambient levels of air pollution in a large metropolitan area. Cornea.

[16] Zhang Y.S., Ma G.X., Yu F., Cao D. (2013). Health damage assessment due to PM_2.5_ exposure during haze pollution events in Beijing-Tianjin-Hebei region in January 2013. Zhonghua Yi Xue Za Zhi.

[17] Gao M., Guttikunda S.K., Carmichael G.R., Wang Y.S., Liu Z.R., Stanier C.O., Saide P.E., Yu M. (2015). Health impacts and economic losses assessment of the 2013 severe haze event in Beijing area. Sci. Total Environ..

[18] Viidanoja J., Sillanpää M., Laakia J., Kerminen V.M., Hillamo R., Aarnio P., Koskentalo T. (2002). Organic and black carbon in PM_2.5_ and PM_10_: 1 year of data from an urban site in Helsinki, Finland. Atmos. Environ..

[19] Alexeeff S.E., Coull B.A., Gryparis A., Suh H., Sparrow D., Vokonas P.S., Schwartz J. (2011). Medium-term exposure to traffic-related air pollution and markers of inflammation and endothelial function. Environ. Health Perspect..

[20] Park S.K., O’Neill M.S., Vokonas P.S., Sparrow D., Rd S.A., Tucker K.L., Suh H., Hu H., Schwartz J. (2008). Traffic-related particles are associated with elevated homocysteine: The VA normative aging study. Am. J. Respir. Crit. Care Med..

[21] Schneider A., Hampel R., Ibald-Mulli A., Zareba W., Schmidt G., Schneider R., Rückerl R., Couderc J.P., Mykins B., Oberdörster G. (2010). Changes in deceleration capacity of heart rate and heart rate variability induced by ambient air pollution in individuals with coronary artery disease. Part. Fibre Toxicol..

[22] Zanobetti A., Schwartz J. (2006). Air pollution and emergency admissions in Boston, MA. J. Epidemiol. Community Health.

[23] Louwies T., Nawrot T., Cox B., Dons E., Penders J., Provost E., Panis L.I., De Boever P. (2015). Blood pressure changes in association with black carbon exposure in a panel of healthy adults are independent of retinal microcirculation. Environ. Int..

[24] Gold D.R., Litonjua A.A., Zanobetti A., Coull B.A., Schwartz J., Maccallum G., Verrier R.L., Nearing B.D., Canner M.J., Suh H. (2005). Air pollution and ST-segment depression in elderly subjects. Environ. Health Perspect..

[25] Wang X., Chen R.J., Meng X., Geng F.H., Wang C.C., Kan H.D. (2013). Associations between fine particle, coarse particle, black carbon and hospital visits in a Chinese city. Sci. Total Environ..

[26] Janssen N.A.H., Hoek G., Simic-Lawson M., Fischer P., van Bree L., ten Brink H., Keuken M., Atkinson R.W., Anderson H.R., Brunekreef B. (2011). Black carbon as an additional indicator of the adverse health effects of airborne particles compared with PM_10_ and PM_2.5_. Environ. Health Perspect..

[27] World Health Organization (WHO) (2015). W.H.O. International Classification of Diseases (ICD-10).

[28] Bateson T.F., Schwartz J. (1999). Control for seasonal variation and time trend in case-crossover studies of acute effects of environmental exposures. Epidemiology.

[29] Lee J.T., Schwartz J. (1999). Reanalysis of the effects of air pollution on daily mortality in Seoul, Korea: A case-crossover design. Environ. Health Perspect..

[30] Ma W.J., Chen R.J., Kan H.D. (2014). Temperature-related mortality in 17 large Chinese cities: How heat and cold affect mortality in China. Environ. Res..

[31] Guo Y.M., Barnett A.G., Pan X.C., Yu W.W., Tong S.L. (2011). The impact of temperature on mortality in Tianjin, China: A case-crossover design with a distributed lag nonlinear model. Environ. Health Perspect..

[32] Winquist A., Kirrane E., Klein M., Strickland M., Darrow L.A., Sarnat S.E., Gass K., Mulholland J., Russell A., Tolbert P. (2014). Joint effects of ambient air pollutants on pediatric asthma emergency department visits in Atlanta, 1998–2004. Epidemiology.

[33] Sarnat S.E., Winquist A., Schauer J.J., Turner J.R., Sarnat J.A. (2015). Fine particulate matter components and emergency department visits for cardiovascular and respiratory diseases in the St. Louis, Missouri-illinois, metropolitan area. Environ Health Perspect..

[34] Krzyzanowski M., Kunadibbert B., Schneider J., Krzyzanowski M., Kunadibbert B., Schneider J. (2005). Health effects of transport-related air pollution. Health Effects Transport-Relat. Air Pollut..

[35] Torricelli A.A.M., Matsuda M., Novaes P., Braga A.L.F., Saldiva P.H.N., Alves M.R., Monteiro M.L.R. (2014). Effects of ambient levels of traffic-derived air pollution on the ocular surface: Analysis of symptoms, conjunctival goblet cell count and mucin 5AC gene expression. Environ. Res..

[36] Szyszkowicz M., Kousha T., Castner J. (2016). Air pollution and emergency department visits for conjunctivitis: A case-crossover study. Int. J. Occup. Med. Environ. Health.

[37] Chu J., Liu L., Li S., Shang K. Analysis of haze pollution in the winter of 2012 in Shijiazhuang. Proceedings of the Climatic and Environment Changes and Human Health (S15), Chinese Meteorological Society Meeting.

[38] Chen S.Y., Lin Y.L., Chang W.T., Lee C.T., Chan C.C. (2014). Increasing emergency room visits for stroke by elevated levels of fine particulate constituents. Sci. Total Environ..

[39] Xu M.M., Jia Y.P., Li G.X., Pan X.C. (2011). Evaluation of personal integrated exposure to fine particle in a community in Beijing. J. Environ. Health.

