# Supplementary material for: Associations of PM2.5 and Black Carbon with Hospital Emergency Room Visits during Heavy Haze Events: A Case Study in Beijing, China
Source: Int J Environ Res Public Health. 2017 Jul 5;14(7):725. doi: 10.3390/ijerph14070725 (PMC5551163; doi:10.3390/ijerph14070725)
Supplement: Supplementary file 1 [file ijerph-14-00725-s001.pdf]

Supplementary Materials: Associations of $PM_{2.5}$ and Black Carbon with Hospital Emergency Room Visits during Heavy Haze Events: A Case Study in Beijing, China

Table S1. Sensitivity analysis on the imputation for missing data of BC in single-pollutant models.

ERVs	Lag days	Percentage increase (95%CI) (%)	
		With missing data of BC	With missing-imputed data of BC
Res.	Lag02	27.6 (6.7, 52.6)	27.6 (9.6, 48.6)
Car.	Lag0	11.6 (-10.5, 39.6)	18.8 (1.4, 39.2)
Ocu.	Lag0	22.1 (3.6, 43.8)	11.8 (-1.4, 26.8)

Table S2. Sensitivity analysis on the adequacy of linear terms of $PM_{2.5}$ or BC in single-pollutant models.

ERVs	Lag days	Percentage increase (95%CI) (%)			
		Linear term of $PM_{2.5}$	Non-linear terms of $PM_{2.5}$	Linear term of BC	Non-linear terms of BC
Res.	Lag02	27.5 (13.0, 43.9)	26.3 (2.7, 55.3)	27.6 (9.6 48.6)	45.0 (17.4, 79.1)
Car.	Lag0	19.4 (2.5, 39.0)	16.7 (−5.8, 44.6)	18.8 (1.4, 39.2)	18.8 (−3.4, 46.0)
Ocu.	Lag0	12.6 (0.0, 26.7)	5.6 (−10.2, 24.2)	11.8 (−1.4, 26.8)	7.2 (−8.7, 25.9)
